# RETRACTED: Gravina et al. The Brain Penetrating and Dual TORC1/TORC2 Inhibitor, RES529, Elicits Anti-Glioma Activity and Enhances the Therapeutic Effects of Anti-Angiogenetic Compounds in Preclinical Murine Models. *Cancers* 2019, *11*, 1604

**DOI:** 10.3390/cancers18010122

**Published:** 2025-12-30

**Authors:** Giovanni Luca Gravina, Andrea Mancini, Alessandro Colapietro, Simona Delle Monache, Roberta Sferra, Simona Pompili, Flora Vitale, Stefano Martellucci, Francesco Marampon, Vincenzo Mattei, Leda Biordi, David Sherris, Claudio Festuccia

**Affiliations:** 1 Department of Biotechnological and Applied Clinical Sciences, Division of Radiation Oncology, University of L’Aquila, 67100 L’Aquila, Italy; giovanniluca.gravina@univaq.it; 2 Department of Biotechnological and Applied Clinical Sciences, Laboratory of Radiobiology, University of L’Aquila, 67100 L’Aquila, Italy; mancio_1982@hotmail.com (A.M.); alecolapietro@gmail.com (A.C.); 3 Department of Biotechnological and Applied Clinical Sciences, Laboratory of Cellular Biology, University of L’Aquila, 67100 L’Aquila, Italy; simona.dellemonache@univaq.it (S.D.M.); s.martellucci@sabinauniversitas.it (S.M.); 4 Department of Biotechnological and Applied Clinical Sciences, Laboratory of Human Anatomy, University of L’Aquila, 67100 L’Aquila, Italy; roberta.sferra@univaq.it (R.S.); pompili.simona@virgilio.it (S.P.); 5 Department of Biotechnological and Applied Clinical Sciences, Laboratory of Neurophysiology, University of L’Aquila, 67100 L’Aquila, Italy; floravitale86@hotmail.it; 6 Laboratory of Experimental Medicine and Environmental Pathology, University Hub “Sabina Universitas”, 02100 Rieti, Italy; vincenzo.mattei@uniroma1.it; 7 Department of Radiological, Oncological and Pathological Sciences, Sapienza University of Rome, 00118 Rome, Italy; francesco.marampon@uniroma1.it; 8 Department of Biotechnological and Applied Clinical Sciences, Laboratory of Experimental Oncology, University of L’Aquila, 67100 L’Aquila, Italy; leda.biordi@univaq.it; 9 Sherris Pharma Partners, Jamaica Plain, MA 02130, USA; davidsherris@gmail.com

The journal retracts the article, “The Brain Penetrating and Dual TORC1/TORC2 Inhibitor, RES529, Elicits Anti-Glioma Activity and Enhances the Therapeutic Effects of Anti-Angiogenetic Compounds in Preclinical Murine Models” [1], cited above.

Following publication, concerns were brought to the attention of the Editorial Office regarding the duplication or partial overlap between a sub-image in Figure 6 [1] and sub-images in Figure 5 in another publication [2] with partially overlapping authorship.

Adhering to our standard procedure, an investigation was conducted by the Editorial Office and the Editorial Board that confirmed two partial overlaps and one duplication between Figure 6 in this publication [1] and sub-figures included in Figure 6 in another paper [2]. While the authors provided replacement images during the investigation, the Editorial Board found the explanations regarding the duplicated data insufficient to warrant a correction. Consequently, the Editorial Board has lost confidence in the reliability of the findings and decided to retract this publication [1], as per MDPI’s retraction policy (https://www.mdpi.com/ethics#_bookmark30).

This retraction was approved by the Editor-in-Chief of the *Cancers* journal.

The authors did not provide a comment on this decision.

The corresponding author, Claudio Festuccia, takes full responsibility for the concerns raised and clarifies that the other co-authors were not directly involved in the duplication of the figures in question.
